# Impact of the Spinal Instability Neoplastic Score on Postoperative Prognosis in Patients with Metastatic Cancer of the Cervical Spine

**DOI:** 10.3390/jcm13247860

**Published:** 2024-12-23

**Authors:** Dong-Ho Kang, Kyunghun Jung, Jin-Sung Park, Minwook Kang, Chong-Suh Lee, Se-Jun Park

**Affiliations:** 1 Department of Orthopedic Surgery, Samsung Medical Center, Seoul 06351, Republic of Koreailucky7ik@gmail.com (K.J.); npng4eve@gmail.com (M.K.); 2 Department of Orthopedic Surgery, Haeundae Bumin Hospital, Busan 48094, Republic of Korea

**Keywords:** metastatic cancer of the cervical spine, Spinal Instability Neoplastic Score, overall survival, prognostic factors, surgical outcomes, spinal instability

## Abstract

**Background:** Although the Spinal Instability Neoplastic Score (SINS) is widely utilized to evaluate spinal instability, its prognostic value for survival in patients with cervical spinal metastases remains unclear. This study investigated the association between the SINS and survival outcomes in patients with metastatic cervical spine cancer. **Methods:** This retrospective cohort study included 106 patients who underwent surgery for metastatic cervical spine cancer at a single institution between 1995 and 2023. Patients were divided into two groups: high SINS (≥13) and low-to-moderate SINS (0–12). Overall survival (OS) was the primary outcome and was analyzed using Kaplan–Meier estimates and Cox regression. Secondary outcomes included changes in Eastern Cooperative Oncology Group Performance Status (ECOG-PS), operation time, estimated blood loss, and postoperative complications. **Results:** The median OS was significantly shorter in the high SINS group compared to the low-to-moderate SINS group (5.3 months versus 8.6 months; *p* = 0.023). A high SINS was independently associated with increased mortality risk (hazard ratio [HR], 1.959; 95% CI, 1.221–3.143; *p* = 0.005). Lung cancer (HR, 4.004; 95% CI, 1.878–8.535; *p* < 0.001) and rectal cancer (HR, 3.293; 95% CI, 1.126–9.632; *p* = 0.029) were predictive of worse survival, whereas postoperative chemotherapy (HR, 0.591; 95% CI, 0.381–0.917; *p* = 0.019) and radiotherapy (HR, 0.531; 95% CI, 0.340–0.827; *p* = 0.005) were associated with improved survival. Changes in the ECOG-PS and postoperative complication rates were not significantly different between the groups. **Conclusions:** A high SINS was associated with significantly shorter survival in patients with metastatic cervical spine cancer, reflecting both mechanical instability and tumor aggressiveness.

## 1. Introduction

Metastatic cancer of the cervical spine is the third most common site of spinal metastasis after the thoracic and lumbar spine and presents unique challenges due to the distinct anatomical and biomechanical characteristics of the cervical spine [1,2,3,4,5]. The cervical spine’s lack of rib support and high mobility increases its susceptibility to instability [6]. In this context, evaluating spinal stability in metastatic cancer of the cervical spine is crucial not only for preventing catastrophic events such as neurological compromise, but also for planning effective cervical-specific surgical and non-surgical treatments [7].

The Spinal Instability Neoplastic Score (SINS) has been widely used to assess the stability of spinal metastases [8]. This score was initially developed using the modified Delphi technique, focusing solely on spinal instability, and has therefore been traditionally regarded as unrelated to survival outcomes [9], with studies reporting no real associations [10,11,12]. For this reason, many prognostic scoring systems for spinal metastases exclude SINS [13,14,15,16]. However, a recent study suggested that a high SINS may be associated with poor prognosis [17], challenging the initial understanding of its prognostic value.

Although the prognostic value of the SINS remains controversial, its potential implications for metastatic cancer of the cervical spine remain unknown. The unique anatomy and biomechanics of the cervical spine affect both surgical approaches and outcomes, highlighting the need for focused studies on the prognostic value of the SINS. Therefore, this study sought to address this gap by examining the surgical outcomes in patients with high and low SINS. We compared surgical outcomes between high SINS groups (≥13) and low-to-moderate SINS groups (0–12), evaluating whether SINS can serve as a meaningful predictor of prognosis and other treatment outcomes.

## 2. Materials and Methods

### 2.1. Study Design and Ethical Considerations

This retrospective cohort study was conducted at a single tertiary referral center specializing in spinal oncology. Data were obtained from the center’s prospective Spinal Metastasis Registry. The study protocol was reviewed and approved by the Institutional Review Board (IRB) of Samsung Medical Center (IRB Approval Number: 2024-11-070). The requirement for informed consent was waived due to the retrospective nature of the study. Patient confidentiality was maintained by anonymizing the data and adhering to the principles outlined in the Declaration of Helsinki. Data were collected from electronic medical records and patient’s picture archiving and communication systems.

### 2.2. Participants

Patients who underwent surgical treatment for metastatic cervical cancer at our institution between January 1995 and December 2023 were included in this retrospective cohort study. Eligible participants were identified using an institutional electronic medical records system. The inclusion criteria were as follows: (1) spinal metastasis diagnosed between 1995 and 2023 and (2) surgical treatment specifically for cervical spinal metastasis. The exclusion criteria were as follows: (1) incomplete or inaccurate clinical and radiological data necessary for SINS evaluation and (2) death due to non-tumor-related causes. The patients were categorized into two groups based on their SINS: high and low-to-moderate.

### 2.3. Outcome Measures and Data Collection

The primary outcome was overall survival (OS), which was defined as the time from surgery to death from any cause, with surviving patients censored at the last follow-up. Secondary outcomes included postoperative changes in the Eastern Cooperative Oncology Group-Performance Status (ECOG-PS), surgical burden including operation time, estimated blood loss, postoperative complications, and independent predictors of survival identified through Cox regression analysis.

Patient demographic and baseline characteristics, including age, sex, primary cancer type, ECOG-PS, Frankel grade, and Karnofsky Performance Status (KPS), were retrospectively extracted. Tumor-specific variables, such as SINS, number of extraspinal bony metastases, visceral metastases, and primary location of cervical lesions, were recorded along with preoperative and postoperative therapeutic interventions, including chemotherapy and radiotherapy.

Surgical variables documented included operation type (e.g., anterior debulking and fixation, posterior debulking and fixation, and fixation), operation time, and estimated blood loss. Anterior debulking and fixation included procedures performed using either an anterior only or combined anterior and posterior approach. Complications, including wound infections, fractures, and tumor relapse-associated neurological deterioration, were recorded. Postoperative functional changes were assessed by comparing preoperative and postoperative ECOG-PS scores and categorizing the patients into improved, unchanged, or aggravated groups.

### 2.4. Statistical Analysis

Continuous variables are expressed as means with standard deviations or medians with interquartile ranges, as appropriate. Categorical variables are presented as frequencies and percentages. Comparisons between the high and low-to-moderate SINS groups were conducted using the independent two-sample *t*-test or Mann–Whitney U test for continuous variables and the chi-square test or Fisher’s exact test for categorical variables. Survival outcomes were analyzed using Kaplan–Meier survival estimates, and the log-rank test was used to compare survival distributions between the groups. Median OS was reported with a 95% confidence interval (CI). Univariate and multivariate Cox proportional hazards regression analyses were conducted to identify independent predictors of survival. Variables with *p*-values < 0.10 in univariate analysis were included in the multivariate model. Hazard ratios (HRs) with 95% CIs were calculated to quantify the strength of the association. Postoperative changes in ECOG-PS were analyzed using paired comparisons of pre- and postoperative scores within each group using chi-squared analysis. Statistical analyses were performed using SPSS (version 27.0; SPSS Inc., Chicago, IL, USA), and statistical significance was set at *p* < 0.05.

## 3. Results

### 3.1. Participant Characteristics and Comparison of Low-to-Moderate and High SINS Groups

Ultimately, 106 patients who underwent surgical treatment for metastatic cervical cancer between January 1995 and December 2023 were included in this study. A total of 71 patients (67.0%) were allocated to the low-to-moderate SINS group and 35 (33.0%) to the high SINS group. The baseline characteristics of the patients are summarized in Table 1. The mean age of all participants was 58.2 ± 10.1 years. Male patients comprised 57.5% of the cohort. Lung cancer (26.4%) and liver cancer (19.8%) were the most common primary tumor types, followed by breast cancer (12.3%), colorectal cancer (5.7%), and kidney cancer (4.7%). There were no significant differences between the two groups in terms of age, sex, or primary tumor type. Furthermore, measures of functional and performance statuses, including ECOG-PS, Frankel grade, and KPS, were comparable between the groups. Other characteristics, such as the number of vertebral body metastases, presence of visceral metastases, number of extraspinal bony metastases, and perioperative chemotherapy and radiotherapy, also showed no significant differences. Similarly, the main locations of the cervical lesions were evenly distributed between the groups. Significant differences were observed between surgical approaches. Surgical strategies differed between the groups, with patients with high SINS undergoing anterior debulking more frequently than those with low to moderate SINS; however, the difference was not significant (71.4% vs. 49.3%; *p* = 0.105).

### 3.2. Survival Analysis

**Figure 1:**
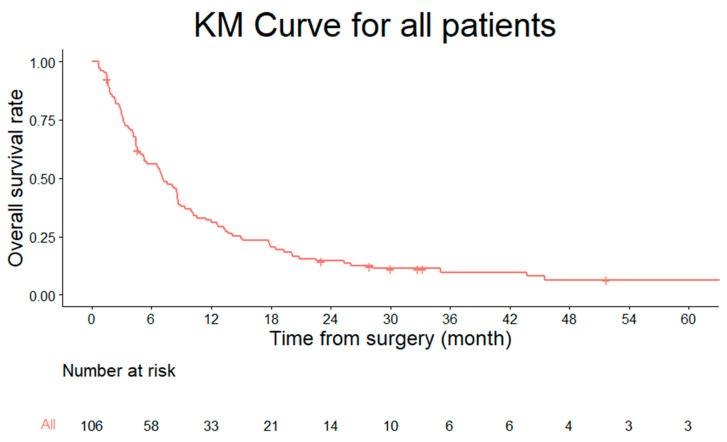
Kaplan–Meier survival curve showing the overall survival of 106 patients with metastatic cervical spine cancer.

**Figure 2:**
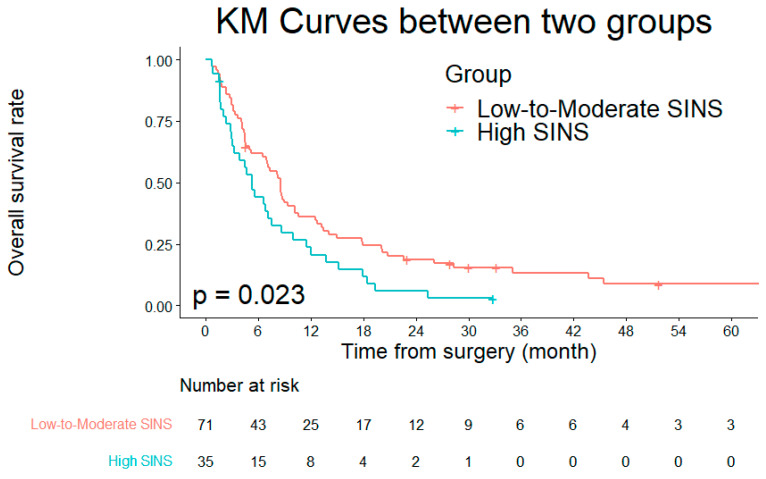
Kaplan–Meier survival curve comparing overall survival between the low-to-moderate SINS group (median survival: 8.6 months) and the high SINS group (median survival: 5.3 months; *p* = 0.023).

The median estimated survival for all patients was 7.1 months (95% CI, 5.3–8.9) (Figure 1). Kaplan–Meier survival analysis demonstrated a significantly shorter median survival in the high SINS group (5.3 months; 95% CI, 3.8–6.8) compared to the low-to-moderate SINS group (8.6 months; 95% CI, 6.9–10.2; *p* = 0.023) (Figure 2).

### 3.3. Changes in Functional Status

In the low-to-moderate SINS group, 18.3% of patients revealed improvements in ECOG-PS, 53.5% showed no change, and 26.8% experienced worsening of their performance status (Table 2). Similarly, in the high SINS group, 17.1% of the patients demonstrated improvement, 54.3% showed no change, and 28.6% had worsening ECOG-PS (Table 3). There was no significant difference between the two groups in the proportion of patients with improved ECOG-PS after surgery (*p* = 0.883) or those with worsening ECOG-PS (*p* = 0.844).

### 3.4. Surgical Burden and Postoperative Complications

**Figure 3:**
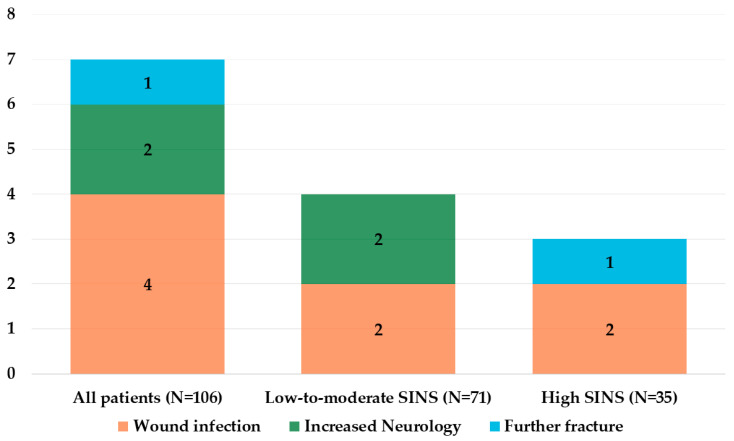
Surgical complications across the low-to-moderate and high SINS groups.

The mean operation time for all patients was 5.6 ± 2.2 h, with the low-to-moderate SINS group having a longer mean operation time compared to the high SINS group (5.9 ± 2.5 h vs. 5.0 ± 1.5 h; *p* = 0.020) (Table 4). However, there was no significant difference in the estimated blood loss between the two groups (low-to-moderate SINS: 684.5 ± 1215.9 mL vs. high SINS: 553.4 ± 473.4 mL; *p* = 0.541). Postoperative complications were comparable between the groups (Figure 3). The overall rate of revision surgeries due to complications was low in both groups, with four patients (5.6%) in the low-to-moderate SINS group and three patients (8.6%) in the high SINS group requiring additional interventions (*p* = 0.682). The overall rate of wound infection was 3.8%, with no significant difference between the groups (*p* = 0.597). In the low-to-moderate SINS group, two patients (2.8%) experienced wound infections compared to two patients (5.7%) in the high SINS group. Further fractures were rare, occurring in only one patient (2.9%) in the high SINS group, whereas no cases were reported in the low-to-moderate SINS group (*p* = 0.330). Tumor relapse-associated neurological deterioration was noted in two patients (2.8%) in the low-to-moderate SINS group, whereas no cases occurred in the high SINS group (*p* = 0.999).

### 3.5. Cox Regression Analysis

In the multivariate analysis, a high SINS was identified as an independent predictor of increased mortality risk (HR, 1.959; 95% CI, 1.221–3.143; *p* = 0.005) (Table 5). Specific primary tumor origins included lung cancer (hazard ratio [HR], 4.004; 95% [CI], 1.878–8.535; *p* < 0.001) and rectal cancer (HR, 3.293; 95% CI, 1.126–9.632; *p* = 0.029). Conversely, postoperative chemotherapy (HR, 0.591; 95% CI, 0.381–0.917; *p* = 0.019) and radiotherapy (HR, 0.531; 95% CI, 0.340–0.827; *p* = 0.005) were independently associated with a reduced mortality risk.

## 4. Discussion

This study highlights the prognostic significance of the SINS in patients with metastatic cervical spine cancer. Our findings show that a high SINS is independently associated with reduced overall survival, with patients in this group exhibiting a median survival of only 5.3 months compared with 8.6 months in the low-to-moderate SINS group. Conversely, postoperative chemotherapy and radiotherapy were shown to improve survival.

SINS was initially developed to predict spinal instability requiring surgical fixation rather than patient survival. Our findings suggest that SINS may have additional prognostic value in patients with metastatic cervical spine cancer due to its unique biomechanical considerations. In the literature, the relationship between SINS and survival outcomes has been debated. Previous studies have reported conflicting results regarding the prognostic value [10,11,12,17]. Ha et al. reported no direct impact of SINS on survival in metastatic lung and hepatocellular cancers, emphasizing the importance of performance status and systemic treatment [10]. Zadnik et al. found no significant association between SINS and survival in patients undergoing surgery for multiple myeloma, indicating that SINS primarily reflects mechanical instability rather than tumor aggressiveness [12]. However, their study reported that the mean SINS was higher in patients who survived more than 1 year compared to those who survived less than 1 year (11 ± 2.6 months vs. 8.5 ± 2.4 months; *p* = 0.056). They also showed that the Kaplan–Meier survival curves for indeterminate stability (SINS 7–12) and instability (SINS 13–18) were clearly separated. However, the difference in survival between the two groups was not statistically significant based on the log-rank test (*p* = 0.12). Although the difference was not statistically significant, this may be attributed to the small sample size (31 patients). Another study by Zadnik assessed an observational cohort of 43 patients who underwent surgical resection for metastatic breast cancer and investigated the impact of SINS [11]. In this analysis, there was no statistically significant difference in survival between patients with SINSs indicating indeterminate stability (7–12) versus gross instability (13–18). However, the median survival was only 12.7 months in the unstable group compared to 28.1 months for the indeterminately stable groups, respectively. The groups were similar in terms of the surgical level, preoperative KPS, and patient age. Importantly, the SINS data were based on 22 patients, and the lack of statistical significance may be attributable to the small sample size.

However, recent studies have challenged these findings. Versteeg et al. reviewed the use of the SINS in clinical practice and identified studies suggesting a potential link between higher SINSs and poorer outcomes, particularly in patients with high systemic tumor burdens [9]. Similarly, Miyaji et al. evaluated the SINS in castration-resistant prostate cancer and revealed that patients with unstable spines (SINS ≥ 7) had a significantly reduced survival compared to those with stable spines (SINS ≤ 6), with a hazard ratio of 2.60 (95% CI, 1.07–5.93; *p* = 0.0345), which is echoed by our result [17]. A possible explanation for these results is the hypothesis that high SINSs may not only signify mechanical instability but also reflect the biological aggressiveness of the tumor and a higher predisposition for systemic spread. Patients with high SINSs in our cohort exhibited a significantly shorter median survival (5.3 months) compared to those with low-to-moderate scores (8.6 months), reinforcing the prognostic relevance of this tool in this specific patient population. This finding underscores the dual role of the SINS as both a stability assessment tool and an indicator of tumor severity, particularly in the context of cervical spine metastases. Cervical lesions are more prone to instability than thoracic lesions, which are supported by ribs. As a result, the onset of symptoms prompting surgery is likely to be similar between the low and high SINS groups. Therefore, a higher SINS, which reflects greater bone destruction at the time of surgery, may indicate more aggressive tumor characteristics. The SINS may also serve as a complementary tool in prognostic evaluations for patients with metastatic cervical spine cancer. A high SINS can suggest a prognosis that is potentially worse than what previous legacy prognostic models predicted. This should be taken into account when assessing the suitability of surgical intervention and in choosing the appropriate surgical method. Furthermore, surgeons and oncologists could consider integrating the SINS into existing prognostic systems or utilizing it to develop new scoring models tailored specifically to cervical spine metastases.

Interestingly, the proportion of patients with unchanged ECOG-PS was higher than that of patients with improvement or worsening, indicating that while surgery may stabilize functional status, achieving substantial recovery is challenging. Specifically, 28.6% of patients in the high SINS group and 26.8% in the low-to-moderate SINS group had deteriorated ECOG-PS, whereas only 17.1% and 18.3%, respectively, demonstrated improvement. This aligns with the findings of Moon et al., who observed that spinal decompression surgery often prevents further neurological decline but does not guarantee significant functional improvement, particularly in advanced cancer [18].

In Table 4, the operation time was slightly longer in the low-to-moderate SINS group (*p* = 0.020). This difference may be attributed to the relatively higher proportion of C2 lesions in the low-to-moderate SINS group, as C2 lesions typically require more complex surgical approaches. However, from a clinical perspective, this difference in operation time is not considered to be of significant importance, as it did not influence other perioperative outcomes or overall survival. Estimated blood loss and perioperative complication rates were comparable between the two groups, except for operation time. This similarity likely reflects that the choice of surgical approach in cervical lesions is typically determined by the tumor’s location (anterior or posterior) rather than the SINS. As a result, both groups underwent similar surgical procedures, which may explain the lack of significant differences in surgical burden and perioperative complications. In our study, the total adverse event rate was 8.5%, which was notably lower than the rates reported in other studies on surgical interventions for spinal metastases. Lau et al. reported an overall complication rate of 21.7% in a cohort of patients undergoing surgery for spinal metastasis [19]. Tan et al. also reported a 20.7% surgical complication rate in patients who underwent surgery for spinal metastases [20]. This disparity can be explained by the influence of the survival duration on the reported incidence of postoperative complications. In a study by Tan et al., the median survival of all patients was 16 months, allowing a longer window for the development of postoperative complications. Conversely, the median survival in our cohort was only 7.1 months, which was significantly shorter and likely limited the time frame for adverse events to occur. Further research is required to clarify whether patients with metastatic cervical cancer have inferior survival rates after surgical treatment.

This study has several limitations. First, its retrospective design inherently introduced a selection bias, as only patients who underwent surgical treatment were included. This may limit the generalizability of the findings to patients managed with nonsurgical treatments or those with less severe disease. Second, the study was conducted at a single tertiary referral center, which may have restricted the applicability of the results to other institutions with different patient populations and management practices. Third, the relatively small sample size, particularly in the high SINS group, may have limited the statistical power to detect subtle differences in secondary outcomes such as functional status changes and complication rates. Fourth, there is a possibility of survivor bias. As only patients who underwent surgery were included, those with more severe conditions who were ineligible for surgery may be underrepresented. This could have influenced survival and outcome findings. Fifth, there is no consideration of benign aggressive tumors, such as giant cell tumors, which can also cause instability. For giant cell tumors, aggressive surgical interventions and denosumab therapy are essential for achieving favorable outcomes [21]. Future studies should include these types of tumors. Furthermore, the use of historical data spanning nearly three decades (1995–2023) introduces potential variability in surgical techniques, perioperative care, and adjunctive therapies that may influence patient outcomes. For instance, the introduction of targeted chemotherapy has revolutionized the treatment landscape for metastatic cancer, offering more effective systemic control and potentially influencing survival outcomes. While stratified analysis by decade or surgical technique could provide valuable insights, the limited sample size in each decade would likely result in insufficient statistical power for meaningful results. Therefore, future prospective studies should aim to validate these findings in larger multicenter cohorts to reduce bias and enhance the reliability of the results.

## 5. Conclusions

This study demonstrated that high SINSs (≥13) in patients with metastatic cervical spine cancer were associated with poorer survival outcomes. In these patients, SINS can serve as a dual-purpose tool, highlighting mechanical instability and providing insights into the biological behavior of the tumor. Patients with high SINSs had a significantly shorter median survival (5.3 months) than those in the low-to-moderate SINS group (8.6 months). These findings support the integration of the SINS into patient management strategies, either as a complementary tool for prognostic evaluations or as part of novel scoring systems, to improve prognostic accuracy and guide treatment decisions. Future studies should aim to validate these findings in larger, multicenter cohorts and explore the integration of SINS into comprehensive prognostic models.

## Figures and Tables

**Table 1:** Baseline characteristics of all patients and their comparison across the low-to-moderate and high SINS groups.

Characteristics	All Patients (N = 106)	Low-to-Moderate SINS (N = 71)	High SINS (N = 35)	*p*-Value
Age, year, mean ± SD	58.2 ± 10.1	58.9 ± 9.7	56.8 ± 10.9	0.321
Male, n (%)	61 (57.5)	44 (62.0)	17 (48.6)	0.189
Primary cancer, n (%)				0.086
Lung	28 (26.4)	21 (29.6)	7 (20.0)	
Liver	21 (19.8)	17 (23.9)	4 (11.4)	
Breast	13 (12.3)	6 (8.5)	7 (20.0)	
Colorectal	6 (5.7)	5 (7.0)	1 (2.9)	
Kidney	5 (4.7)	4 (5.6)	1 (2.9)	
Prostate	2 (1.9)	2 (2.8)	0 (0.0)	
Thyroid	2 (1.9)	2 (2.8)	0 (0.0)	
Others	29 (27.4)	14 (19.7)	15 (42.9)	
ECOG-PS, n (%)				0.559
0	2 (1.9)	1 (1.4)	1 (2.9)	
1	40 (37.7)	30 (42.3)	10 (28.6)	
2	35 (33.0)	22 (31.0)	13 (37.1)	
3	23 (21.7)	15 (21.1)	8 (22.9)	
4	6 (5.7)	3 (4.2)	3 (8.6)	
Frankel grade, n (%)				0.999
E	48 (45.3)	32 (45.1)	16 (45.7)	
C and D	56 (52.8)	37 (52.1)	19 (54.3)	
A and B	2 (1.9)	2 (2.8)	0 (0.0)	
Karnofsky performance status, n (%)				0.841
Good (80–100%)	34 (32.1)	24 (33.8)	10 (28.6)	
Moderate (50–70%)	53 (50.0)	35 (49.3)	18 (51.4)	
Poor (10–40%)	19 (17.9)	12 (16.9)	7 (20.0)	
Number of extraspinal bony metastases, n (%)				0.572
0	50 (47.2)	31 (43.7)	19 (54.3)	
1–2	23 (21.7)	17 (23.9)	6 (17.1)	
≥3	33 (31.1)	23 (32.4)	10 (28.6)	
Metastasis to visceral organs, n (%)				0.999
No metastases	56 (52.8)	38 (53.5)	18 (51.4)	
Removable	5 (4.7)	3 (4.2)	2 (5.7)	
Unremovable	45 (42.5)	30 (42.3)	15 (42.9)	
Number of metastases in the vertebral body, n (%)				0.345
1	32 (30.2)	22 (31.0)	10 (28.6)	
2	26 (24.5)	20 (28.2)	6 (17.1)	
≥3	48 (45.3)	29 (40.8)	19 (54.3)	
Main cervical lesion				0.482
C1	1 (0.9)	1 (1.4)	0 (0.0)	
C2	18 (17.0)	15 (21.1)	3 (8.6)	
C3	9 (8.5)	7 (9.9)	2 (5.7)	
C4	20 (18.9)	12 (16.9)	8 (22.9)	
C5	15 (14.2)	10 (14.1)	5 (14.3)	
C6	21 (19.8)	11 (15.5)	10 (28.6)	
C7	22 (20.8)	15 (21.1)	7 (20.0)	
SINS				<0.001
0–6	7 (6.6)	7 (9.9)	0 (0.0)	
7–12	64 (60.4)	64 (90.1)	0 (0.0)	
≥13	35 (33.0)	0 (0.0)	35 (100.0)	
Operation type, n (%)				0.105
Fixation only	15 (14.2)	12 (16.9)	3 (8.6)	
Posterior debulking and fixation	31 (29.2)	24 (33.8)	7 (20.0)	
Anterior debulking and fixation	60 (56.6)	35 (49.3)	25 (71.4)	
Usage of occiput plate	8 (7.5)	6 (8.5)	2 (5.7)	0.999
Preoperative chemotherapy, n (%)	56 (52.8)	36 (50.7)	20 (57.1)	0.532
Preoperative radiotherapy, n (%)	38 (26.4)	24 (33.8)	14 (40.0)	0.532
Postoperative chemotherapy, n (%)	50 (47.2)	33 (46.5)	17 (48.6)	0.839
Postoperative radiotherapy, n (%)	64 (60.4)	45 (63.4)	19 (54.3)	0.368

**Table 2:** The number of patients showing postoperative changes in ECOG-PS after surgery in the low-to-moderate SINS group.

Preoperative ECOG-PS	Postoperative ECOG-PS						Total
	0	1	2	3	4	5	
0	0	1 ^b^	0 ^b^	0 ^b^	0 ^b^	0 ^b^	1
1	1 ^a^	17	7 ^b^	3 ^b^	1 ^b^	1 ^b^	30
2	0 ^a^	2 ^a^	15	5 ^b^	0 ^b^	0 ^b^	22
3	0 ^a^	1 ^a^	6 ^a^	7	1 ^b^	0 ^b^	15
4	0 ^a^	0 ^a^	1 ^a^	2 ^a^	0	0 ^b^	3
Total	1	21	29	17	2	1	71

Light grey box ^a^: improved ECOG-PS; white box: no change in ECOG-PS; dark grey box ^b^: aggravated ECOG-PS; SINS, Spinal Instability Neoplastic Scale; ECOG-PS, Eastern Cooperative Oncology Group-Performance Status.

**Table 3:** The number of patients demonstrating postoperative changes in ECOG-PS after surgery in the high SINS group.

Preoperative ECOG-PS	Postoperative ECOG-PS						Total
	0	1	2	3	4	5	
0	1	0 ^b^	0 ^b^	0 ^b^	0 ^b^	0 ^b^	1
1	0 ^a^	5	4 ^b^	1 ^b^	0 ^b^	0 ^b^	10
2	0 ^a^	1 ^a^	9	2 ^b^	1 ^b^	0 ^b^	13
3	0 ^a^	0 ^a^	4 ^a^	3	1 ^b^	0 ^b^	8
4	0 ^a^	0 ^a^	0 ^a^	1 ^a^	1	1 ^b^	3
Total	1	6	17	7	3	1	35

Light grey box ^a^: improved ECOG-PS; white box: no change in ECOG-PS; dark grey box ^b^: aggravated ECOG-PS; SINS, Spinal Instability Neoplastic Scale; ECOG-PS, Eastern Cooperative Oncology Group-Performance Status.

**Table 4:** Surgical burden and perioperative complications of all patients and their comparison across the low-to-moderate and high SINS groups.

	All Patients (N = 106)	Low-to-Moderate SINS (N = 71)	High SINS (N = 35)	*p*-Value
Surgical variables				
Operation time (h)	5.6 ± 2.2	5.9 ± 2.5	5.0 ± 1.5	0.020
Estimated blood loss (mL)	641.2 ± 1030.5	684.5 ± 1215.9	553.4 ± 473.4	0.541
Surgical complications				
Total events requiring revision surgery, n (%)	7 (6.6)	4 (5.6)	3 (8.6)	0.682
Wound infection, n (%)	4 (3.8)	2 (2.8)	2 (5.7)	0.597
Increased neurology due to tumor relapse, n (%)	2 (1.9)	2 (2.8)	0 (0.0)	0.999
Further fracture, n (%)	1 (0.9)	0 (0.0)	1 (2.9)	0.330

**Table 5:** Cox regression analysis identifying factors associated with mortality.

	Univariate Analysis		Multivariate Analysis	
	Hazard Ratio (95% CI)	*p* Value	Hazard Ratio (95% CI)	*p* Value
Sex (male)		0.086		0.430
Low-to-moderate SINS	Reference			
High SINS	1.637 (1.064–2.517)	0.025	1.959 (1.221–3.143)	0.005
Preoperative Frankel grade	0.639 (0.429–0.950)	0.026		0.084
Modified Tokuhashi score	0.923 (0.847–1.006)	0.067		0.838
Primary cancer group of the modified Tokuhashi score		0.003		0.008
5 (thyroid, breast, prostate, carcinoid tumor)	Reference			
4 (rectum)	3.049 (1.099–8.459)	0.032	3.293 (1.126–9.632)	0.029
3 (kidney, uterus)	1.314 (0.417–4.138)	0.641		0.337
2 (other)	3.154 (1.556–6.394)	0.001	2.648 (1.295–5.415)	0.008
1 (liver, gallbladder)	2.636 (1.240–5.605)	0.012	2.715 (1.227–6.011)	0.014
0 (lung, pancreas, etc.)	3.686 (1.777–7.646)	<0.001	4.004 (1.878–8.535)	<0.001
Preoperative radiotherapy	1.866 (1.217–2.860)	0.004		0.881
Postoperative chemotherapy	0.477 (0.314–0.725)	0.001	0.591 (0.381–0.917)	0.019
Postoperative radiotherapy	0.502 (0.330–0.763)	0.001	0.531 (0.340–0.827)	0.005

## Data Availability

Data underlying this article cannot be shared publicly because of the privacy of individuals who participated in the study. The data can be shared by the corresponding authors upon reasonable request.
